# Enhancing the interpretation of genetic observations in KCNQ1 in unselected populations: relevance to secondary findings

**DOI:** 10.1093/europace/euad317

**Published:** 2023-10-28

**Authors:** Valeria Novelli, Trent Faultless, Marina Cerrone, Melanie Care, Martina Manzoni, Sara L Bober, Arnon Adler, Fabio De-Giorgio, Danna Spears, Michael H Gollob

**Affiliations:** Centro Cardiologico Monzino, IRCCS, Via C. Parea 4, Milano, 20138, Italy; Toronto General Hospital Research Institute, University of Toronto, Toronto, Canada; Inherited Arrhythmia Clinic and Heart Rhythm Center, ‘Leon Charney’ Division of Cardiology NYU Grossman School of Medicine, NewYork, NY, USA; Inherited Arrhythmia and Cardiomyopathy Program, Division of Cardiology, University of Toronto, 200 Elizabeth St.Rm 3GW-360, Toronto M5G 2C4, Ontario, Canada; Centro Cardiologico Monzino, IRCCS, Via C. Parea 4, Milano, 20138, Italy; Toronto General Hospital Research Institute, University of Toronto, Toronto, Canada; Toronto General Hospital Research Institute, University of Toronto, Toronto, Canada; Department of Health Care Surveillance and Bioethics, Section of Legal Medicine, Fondazione Policlinico A.Gemelli IRCCS,Università Cattolica del Sacro Cuore, 00168, Rome, Italy; Inherited Arrhythmia and Cardiomyopathy Program, Division of Cardiology, University of Toronto, 200 Elizabeth St.Rm 3GW-360, Toronto M5G 2C4, Ontario, Canada; Toronto General Hospital Research Institute, University of Toronto, Toronto, Canada; Inherited Arrhythmia and Cardiomyopathy Program, Division of Cardiology, University of Toronto, 200 Elizabeth St.Rm 3GW-360, Toronto M5G 2C4, Ontario, Canada; Department of Physiology, University of Toronto, Toronto, Canada

**Keywords:** Long QT syndrome, Topology, Genetic testing, KCNQ1, Variant interpretation, Variant of unknown significance

## Abstract

**Aims:**

Rare variants in the *KCNQ1* gene are found in the healthy population to a much greater extent than the prevalence of Long QT Syndrome type 1 (LQTS1). This observation creates challenges in the interpretation of *KCNQ1* rare variants that may be identified as secondary findings in whole exome sequencing.

This study sought to identify missense variants within sub-domains of the *KCNQ1-*encoded Kv7.1 potassium channel that would be highly predictive of disease in the context of secondary findings.

**Methods and results:**

We established a set of *KCNQ1* variants reported in over 3700 patients with diagnosed or suspected LQTS sent for clinical genetic testing and compared the domain-specific location of identified variants to those observed in an unselected population of 140 000 individuals. We identified three regions that showed a significant enrichment of *KCNQ1* variants associated with LQTS at an odds ratio (OR) >2: the pore region, and the adjacent 5th (S5) and 6th (S6) transmembrane (TM) regions. An additional segment within the carboxyl terminus of Kv7.1, conserved region 2 (CR2), also showed an increased OR of disease association. Furthermore, the TM spanning S5–Pore–S6 region correlated with a significant increase in cardiac events.

**Conclusion:**

Rare missense variants with a clear phenotype of LQTS have a high likelihood to be present within the pore and adjacent TM segments (S5–Pore–S6) and a greater tendency to be present within CR2. This data will enhance interpretation of secondary findings within the *KCNQ1* gene. Further, our data support a more severe phenotype in LQTS patients with variants within the S5–Pore–S6 region.

## Structured Graphical Abstract

**Structured Graphical Abstract:**
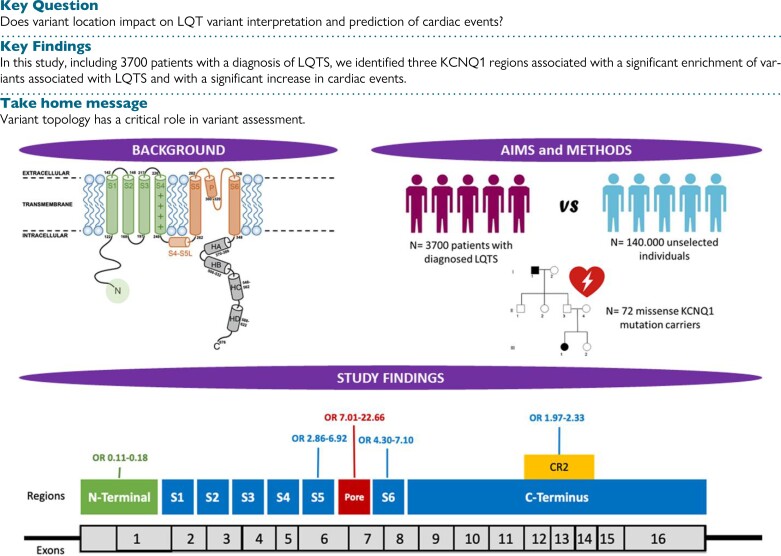

**What’s New?**

- Variant topology has a critical role in the variant assessment.
- This study sought to identify missense variants within sub-domains of the KCNQ1-encoded Kv7.1 potassium channel that would be highly predictive of disease in the context of secondary findings.
- Three KCNQ1 regions are associated with significant enrichment of variants associated with LQTS and an increase in cardiac events.

## Introduction

One of the important lessons learned in recent years in the field of human genetics is that a rare genetic change, even in a gene known to cause human disease does not necessarily represent a pathogenic observation in an individual.^1,2^ This concept necessitates appropriate rules and guidelines to interpret genetic observations, either when made through targeted genetic testing or when observed as a secondary finding when whole exome sequencing (WES) is performed for another condition. Overcalling the relevance of a particular genetic change may have drastic life-changing ramifications for a patient and their family members. Conversely, erring on the side of caution due to limitations in variant interpretation may lead to false reassurance and the absence of cascade screening of family members to guide appropriate clinical surveillance.

In 2015, the American College of Medical Genetics (ACMG) and the Association for Molecular Pathology (AMP) established a framework including a series of stringent criteria to assist in the variant interpretation process.^3^ However, a lack of gene–disease specific guidance decreases the specificity of this approach, thus increasing the number of variants classified as unknown significance (VUS).^4^

Although genetic testing in a selected cohort of patients with suspected disease would be expected to yield a high proportion of rare variants of likely clinical significance, data derived through the UK Biobank indicates that Mendelian genes have a much lower clinical penetrance in unselected cohorts.^5^ This challenges the use of the ClinVar database (ncbi.nlm.nih.gov/clinvar) as the most optimal option for reporting of secondary findings in unselected patients. This issue was previously described by Ghouse *et al*., showing that a proportion of *KCNQ1* missense variants associated with LQT were identified in an unselected Danish population, with normal mean QTc interval and no difference in syncope or overall mortality between carriers and no-carriers.^6^

In the current study, we utilize several large published cohorts of clinically diagnosed LQTS patients and the publicly available gnomAD genetic database (gnomad.broadinstitute.org) representing over 140 000 subjects to serve as an unselected population cohort, to assess whether certain sub-domains of the *KCNQ1*-encoded Kv7.1 channel are more pre-disposed to host rare disease-associated missense variants. Uniquely, we take the novel approach of refining each region, allowing for differences in variant localization that may reflect structural–functional characteristics of the Kv7.1 channel. This approach recognizes, for example, that not all transmembrane (TM) regions may be equal in functional relevance. Lastly, we evaluate whether sub-domain regions more likely to harbour disease-causing variants correlate with cardiac events in a LQTS cohort.

## Methods

### Identification and inclusion criteria of KCNQ1 variants

The *KCNQ1* missense variants identified were derived from 3721 selected patients referred for comprehensive LQTS genetic testing from four different published studies and a clinical LQTS cohort from the Toronto General Hospital Inherited Arrhythmia Registry.^7–10^ The population was distributed as follows: *n* = 2500 patients from Kapplinger *et al*.^7^; *n* = 430 from Napolitano *et al*.^8^; *n* = 388 patients from Kapa *et al*.^9^; *n* = 262 from Splawski *et al*.,^10^ and 141 index cases from the Toronto General Hospital ethics-approved Inherited and Cardiomyopathy Registry (*Table 1*). *KCNQ1* missense variants from an unselected cohort of individuals not enriched for a diagnosis of LQTS were collected from the gnomAD database v2.1.1, which has data from over 141 456 unrelated individuals. In order to improve rigor in our analysis and ensure a topological location-only comparison, we analysed unique variants and not the number of patients with specific variants, avoiding redundant counting of identical variants that may represent population founder mutations or mutation hotspots in LQTS cases. Such variants would not pose challenges to interpretation. We only included variants annotated in gnomAD with a coverage >×20. We restricted our study to only missense variants, thus excluding frame deletions and insertions that can alter the length of the protein, stop-codon, frameshift, and variants affecting the splicing site since these putative loss of function mutations also rarely pose a classification challenge in *KCNQ1-*LQT1, as compared to missense variants that do not predict a truncated protein.

**Table 1:** Patient cohort

Source	Analysed patients	KCNQ1 variants	Missense
Kapa *et al*.^9^	388	87	60
Kapplinger *et al*.^7^	2500	199	133
Splawski *et al*.^10^	262	85	65
Napolitano *et al*.^8^	430	56	42
Toronto Hospital (index cases)	141	47	42

In comparing topological location of variants between LQTS cases and variants from an unselected population cohort, we used multiple comparisons at different minor allele frequency cutoffs. To ensure inclusion of true putative disease-causing *KCNQ1* variants from historical LQTS reported cohorts, we only included variants with a minor allele frequency (MAF) < 10^−4^ and <10^−5^, in separate analyses. The topological location of unique case variants from these two MAF groups were compared to the topological location of all missense variants reported from the unselected gnomAD population (any MAF). Additionally, since LQTS has a disease prevalence of 1/2000, we did further analysis excluding very rare variants (MAF < 10^−4^ and <10^−5^) from the unselected population, recognizing that of 141 000 individuals where these variants were observed that a proportion of very rare variants might represent true LQTS. Lastly, to assess whether our observations of variant distribution between selected and unselected populations was consistent, we further analysed the distribution of only rare variants (MAF < 10^−4^) from both cohorts. In total, topological location of *KCNQ1* variants from a selected LQTS cohort and the unselected population was made in five comparisons: (1) Case variants MAF <10^−4^ vs. unselected population variants (any MAF), (2) case variants < 10^−5^ vs. unselected population variants (any MAF), (3) case variants MAF <10^−4^ vs. unselected population variants MAF >10^−4^, and (4) case variants < 10^−5^ vs. unselected population variants MAF >10^−5^, and (5) case variants MAF <10^−4^ vs. unselected population variants MAF <10^−4^.

A full list of included variants is provided in Supplementary material online, *Table S1*.

### Sub-domain variant carrier status and clinical correlation

To evaluate whether any sub-domain regions enriched for variants in cases correlate with severity of QT prolongation or a history of cardiac events (cardiac arrest or syncope), we reviewed the clinical details of 72 missense *KCNQ1* mutation carriers from 42 unrelated families followed in the Toronto General Hospital Inherited Arrhythmia Registry.

### Kv7.1 channel topology

**Figure 1:**
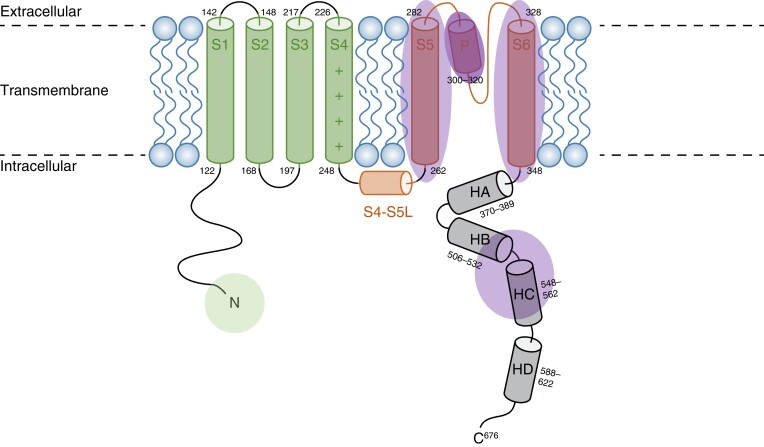
Structural topology of the Kv7.1 channel. Shaded areas indicate regions enriched in case or control variants. N = N-Terminus; C = C-Terminus.

Voltage-gate potassium channels have a typical conserved structure composed of six TM (S1–S6) flanked by intracellular amino (N) and carboxyl (C) terminus regions (*Figure 1*). In the current study, we used UniProt knowledgebase (uniprot.org) to determine the topological and domain-specific location of case and control missense variants.

According to the data reported in UniProt (uniprot.org/uniprot/P51787), *KCNQ1*-encoded Kv7.1 has the following structure: N-terminus (residues 1–121), transmembrane/linker/pore spanning region (TLP, residues 122–348) including the channel pore (residues 300–320) and two connecting cytoplasmic loops (C-loops), and an intracellular C-terminus region (residues 349–676) (*Figure 1*). The TLP and the specific TM regions (S1–S2–S3–S4–S5–S6) were analysed for variant distribution independently to assess for any TM region that may be specifically enriched in case variants reflecting differences in channel structure and function. Specific amino acid boundaries for TM regions S1, S2, S3, S4, S5, and S6, as well as the extracellular linkers and cytoplasmic loops, are provided in Supplementary material online, *Table S2*. Specific structural regions analysed within the C-terminus included: Helix A (residues 370–389), Helix B (residues 506–532), Helix C (residues 548–562), and Helix D (residues 588–622) which also corresponds to the subunit assembly domain (SAD). Lastly, we analysed another three conserved regions (CRs), closely associated with the helical regions. with a high level of conservation among orthologs and paralogs: conserved region 1 (residues 349–391), conserved region 2 (residues 509–575), conserved region 3 (residues 585–607).^11^

### Statistical analysis

Distribution of variants was compared between LQTS cases and variants from the unselected population cohort using Fisher's exact two-tailed test. OR and confidence interval (CI) were calculated as previously described.^12^ Variants present in the first 70 amino acids of KCNQ1 were excluded because of higher risk of sequencing errors in the relevant genomic regions due to lower coverage in the gnomAD database. Results were considered significant when *P*-value <0.05. All statistical comparisons were performed using Stata.

## Results

### Comparison of topological location of selected case and unselect population missense variant distribution in KCNQ1 defines three sub-domains of the TLP as the most intolerant to variation

A total of 561 variants in *KCNQ1* were identified from the LQTS patient cohorts. Among these, 342 were missense (61%) (*Table 1*).

Applying allele frequency thresholds of MAF < 10^−4,^ 190 missense case variants, and all the 311 missense variants (95.8% having a MAF < 10^−4^) from the gnomAD unselected population dataset were used for the initial analysis (*Table 2*).

**Table 2:** Odds ratio for sub-domain localization of case and control variants in Kv7.1

Region	Case variants <10^-4^ vs. all control variants	Case variants <10^-5^ vs. all control variants	Case variants <10^-4^ vs. control variants > 10^-4^	Case variants <10^-5^ vs. controlCSBARLINE variants > 10^-5^	Case variants <10^-4^ vs. control variants < 10^-4^
N-terminus	OR = 0.11; *P* = 0.0002	OR = 0.14; *P* = 0.001	OR = 0.11; *P* = 0.0003	OR = 0.18; *P* = 0.02	OR = 0.11; *P* = 0.0003
S1	OR = 1.65; *P* = 0.52	OR = 2.00; *P* = 0.31	OR = 1.58; *P* = 0.52	OR = 4.34; *P* = 0.22	OR = 1.57; *P* = 0.52
S1–S2 linker	OR = 0.65; *P* = 0.75	OR = 0.78; *P* = 1.00	OR = 0.62; *P* = 0.71	OR = Inf; *P* = 0.50	OR = 0.62; *P* = 0.71
S2	OR = 1.36; *P* = 0.75	OR = 1.32; *P* = 0.74	OR = 1.58; *P* = 0.52	OR = 0.84; *P* = 1.00	OR = 1.57; *P* = 0.52
S2–S3 C-loop	OR = 1.55; *P* = 0.19	OR = 1.71; *P* = 0.12	OR = 1.48; *P* = 0.25	OR = 1.52; *P* = 0.33	OR = 1.48; *P* = 0.25
S3	OR = 1.05; *P* = 1.00	OR = 0.69; *P* = 0.62	OR = 0.96; *P* = 1.00	OR = 0.59; *P* = 0.39	OR = 0.96; *P* = 1.00
S3–S4 linker	OR = 0.81 *P* = 1.00	OR = 0.49; *P* = 0.66	OR = 0.78; *P* = 1.00	OR = 0.42; *P* = 0.59	OR = 0.77; *P* = 1.00
S4	OR = 1.50; *P* = 0.37	OR = 1.45; *P* = 0.46	OR = 1.44; *P* = 0.50	OR = 1.73; *P* = 0.55	OR = 1.44; *P* = 0.49
S4–S5 C-loop	OR = 2.1; *P* = 0.14	OR = 2.28; *P* = 0.11	OR = 2.00; *P* = 1.49	OR = 4.00; *P* = 0.07	OR = 2.00; *P* = 0.14
S5	**OR = 2.86; *P* = 0.01**	**OR = 3.52; *P* = 0.003**	**OR = 3.096; *P* = 0.01**	**OR = 6.92; *P* = 0.004**	**OR = 3.09; *P* = 0.01**
S5-loop	OR = 0.53 *P* = 0.31	OR = 0.16; *P* = 0.07	OR = 0.51; *P* = 0.30	OR = 0.10; *P* = 0.01	OR = 0.51; *P* = 0.30
pore	**OR = 7.30; *P* = 1.9 × 10^−6^**	**OR = 8.66; *P* = 2.6 × 10^−7^**	**OR = 7.01; *P* = 3.02 × 10^−6^**	**OR = 22.66; *P* = 5.4 × 10^−6^**	**OR = 7.01; *P* = 3.2 × 10^−6^**
S6-loop	OR = 1.63; *P* = 0.68	OR = 1.98; *P* = 0.41	OR = 1.57; *P* = 0.68	OR = Inf; *P* = 0.25	OR = 1.57; *P* = 0.68
S6	**OR = 4.49; *P* = 0.02**	**OR = 5.47; *P* = 0.009**	**OR = 4.30; *P* = 0.02**	**OR = 7.10; *P* = 0.04**	**OR = 4.30; *P* = 0.02**
C-terminus	OR = 0.45; *P* = 3.3 × 10^−5^	OR = 0.38; *P* = 2.5 × 10^−6^	OR = 0.47; *P* = 0.0001	OR = 0.32; *P* = 2.44 × 10^−6^	OR = 0.47; *P* = 0.0001

**Figure 2:**
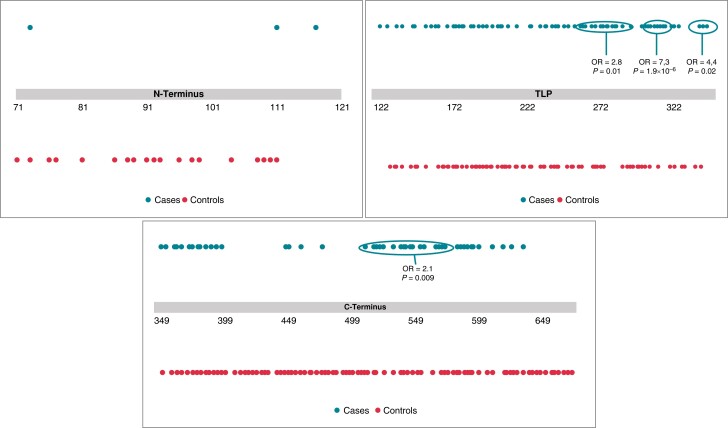
Distribution of *KCNQ1* missense variants identified in cases and presumed healthy controls across the N-terminus. TLP and C-terminus regions of Kv7.1. Blue dots represent the localization of LQTS-associated missense variants found on genetic testing with a MAF < 10^−4^. compared to the distribution of missense variants to those observed in the gnomAD population (orange dots) denote amino acid position.

Comparing the distribution of LQTS-associated missense variants with those present in the unselected population, the TLP spanning from S1 to S6 collectively showed an enrichment of case variants (OR = 3.0; *P* < 0.0001). However, when this analysis was repeatedly analysing each TM region independently, only three TM sub-domains (S5, Pore, and S6) showed a significant enrichment for *KCNQ1* case missense variants (*Figure 2*) (S5: OR = 2.9, *P* < 0.05; Pore: OR = 7.3, *P* < 0.00001; S6: OR = 4.5, *P* < 0.05; combined: OR = 5.3, *P* < 0.000001) while the remaining region of the TLP (S1–S4) showed no significant difference in the frequency of case missense variants as compared to variants present in the unselected population (S1: OR = 1.6, *P* > 0.5; S2: OR = 1.4, *P* > 0.5; S3: OR = 0.9, *P > 1;* S4: OR = 1.5, *P* > 0.3; combined: OR = 1.3, *P* = 0.34). As shown in *Table 2* and *Figure 2*, the pore-forming loop, composed of 20 amino acids (residues 300–320) resulted in the most enriched area of disease-associated missense variants (*Figure 2*). This analysis indicates that the previously accepted premise of the entire TLP (aa 122 to aa 348) representing a high predictive value for a disease-related variant in *KCNQ1*^9,12^ is not accurate when a more domain-specific approach is utilized and in the absence of adding counts of >1 for hot-spot or founder variants, and that the pore loop and adjacent TM domains of S5 and S6 are the only TM regions significantly enriched for *KCNQ1* case missense variants. Thus, 68% of the TLP, representing amino acids 122–261 (S1–S4) and associated linker regions do not show enrichment for *KCNQ1* cases variants. Bold values denote statistical significance .

### A hot-spot area within the KCNQ1 C-terminus

In agreement with other studies,^10^ the conserved region 2 (CR2) of the C-terminus. representing 66 amino acids, was confirmed as an area with significant clustering of disease variants (OR = 2.1, *P* = 0.009) (*Table 3*). However, in contrast with other studies,^8,10^ the remaining sub-regions, including the CR1, CR3, and the SAD did not show a significant difference in the distribution of variants between LQTS patients and the unselected cohort.

**Table 3:** Refined sub-domain localization of case and control variants in Kv7.1

Region	Case variants < 10^-4^ vs. all control variants	Case variants < 10^-5^ vs. all control variants	Case variants < 10^-4^ vs. control variants > 10^-4^	Case variants < 10^-5^ vs. control variants > 10^-5^	Case variants < 10^-4^ vs. control variants < 10^-4^
TLP	**OR = 3.00; *P* = 5.3 × 10^−9^**	**OR = 3.47; *P* = 7.9 × 10^−10^**	**OR = 2.88; *P* = 3.17 × 10^−8^**	**OR = 3.97; *P* = 2.55 × 10^−8^**	**OR = 2.88; *P* = 3.1 × 10^−8^**
TLP without S5–pore–S6	OR = 1.43; *P* = 0.08	OR = 1.36; *P* = 0.14	OR = 1.36; *P* = 0.13	OR = 1.37; *P* = 0.21	OR = 1.49; *P* = 0.05
S5–pore–S6 (TM only)	**OR = 5.31; *P* = 2.4 × 10^−9^**	**OR = 6.68; *P* = 3.1 × 10^−11^**	**OR = 3.11; *P* = 3.70 × 10^−9^**	**OR = 4.74; *P* = 3.3 × 10^−10^**	**OR = 5.41; *P* = 2.43 × 10^−9^**
***C-TERMINAL SUB-REGIONS***					
SAD	OR = 1.35; *P* = 0.64	OR = 0.89; *P* = 1.00	OR = 1.42; *P* = 0.47	OR = 0.84; *P* = 1.00	OR = 1.42; *P* = 0.47
Helix A	OR = 1.28; *P* = 0.61	OR = 1.32; *P* = 0.59	OR = 1.38; *P* = 0.59	OR = 1.02; *P* = 1.00	OR = 1.38; *P* = 0.59
Helix B	OR = 0.91 *P* = 1.00	OR = 0.98; *P* = 1.00	OR = 0.87; *P* = 0.83	OR = 1.13; *P* = 1.00	OR = 0.87; *P* = 0.83
Helix C	OR = 1.65; *P* = 0.39	OR = 0.98; *P* = 1.00	OR = 1.58; *P* = 0.55	OR = 0.84; *P* = 1.00	OR = 1.58; *P* = 0.55
Helix D	OR = 1.05; *P* = 1.00	OR = 0.69; *P* = 0.62	OR = 1.09; *P* = 0.82	OR = 0.51; *P* = 0.27	OR = 1.08; *P* = 082
CR1	OR = 2.05; *P* = 0.06	**OR = 2.23; *P* = 0.004**	OR = 2.11; *P* = 0.06	OR = 2.40; *P* = 0.07	OR = 2.11; *P* = 0.05
CR2	**OR = 2.11 *P* = 0.009**	**OR = 1.97; *P* = 0.002**	**OR = 2.02; *P* = 0.01**	**OR = 2.33; *P* = 0.03**	**OR = 2.02; *P* = 0.01**
CR3	OR = 1.50; *P* = 0.47	OR = 1.18; *P* = 0.79	OR = 1.60; *P* = 0.33	OR = 1.28; *P* = 0.76	OR = 1.59; *P* = 0.33

Bold values denote statistical significance.

CR, conserved region.

Interestingly, our analysis revealed that when the C-terminus is taken as a whole, there is a greater enrichment of variants in the unselected cohort (OR = 0.45, *P* < 0.001), in contrast with previous studies from smaller cohorts suggesting a greater enrichment for case variants^9,11^ (*Table 2*).

Additional analysis, using four other combinations of MAF threshold for cases and unselected individuals, showed consistently the same significant enrichment for *KCNQ1* case missense variants in all the regions identified in the initial analysis. Similarly, the TLP (S1–S4) in the absence of S5, the pore, and S6 did not show an OR of significance (>2.0) or lacked statistical significance (*Table 2*).

### Sub-domain enriched regions and clinical correlation

**Figure 3:**
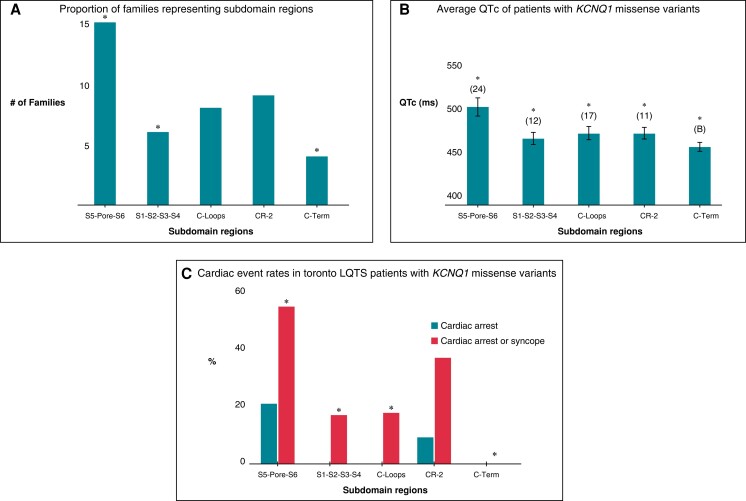
Kv7.1 enriched sub-domain regions and clinical correlation. (*A*) Proportion of families representing subdomain regions. *P < 0.05 vs. S5–Pore–S6; CR2, conserved region 2; C-Term, C-terminal region excluding CR-2. (*B*) Average QTc of patients with KCNQ1 missense variants. Vertical bars represent standard error (SE). (*C*) Cardiac event rates in Toronto LQTS patients with KCNQ1 missense variants. *P < 0.05 vs. S5–Pore–S6.

Following the observations of a significant enrichment for case variants localized within the S5–Pore–S6 region of Kv7.1, we analysed what proportion of index cases followed in the Toronto LQTS Clinic were represented by mutations in this region, and whether S5–Pore–S6 mutation carriers have a greater QTc or history of cardiac events compared to other sub-domains. Of 42 unrelated families with *KCNQ1* missense variants, 15 (36%) were represented by missense mutations within S5–Pore–S6, significantly greater than families with missense mutations in the other specific TM domains combined (S1–S2–S3–S4) (six families,14%) (*P* < 0.05) (*Figure 3A*). Analysis of the first 12 lead ECG from 73 *KCNQ1* missense carriers showed that individuals with mutations localized within S5–Pore–S6 had a significantly greater QTc (501 ± 10.5 m) as compared to other subdomains (S1–S2–S3–S4: QTc 465 ± 6.9 m), (C-loops: QTc 472 ± 7.7 ms), CR-2 (471 ± 6.6 ms), and C-term (456 ± 5.0 ms) (*P* < 0.03 for each comparison vs. S5–Pore–S6) (*Figure 3B*). Of those with S5–Pore–S6 variants, 5 (21%) had a history of cardiac arrest. In comparing any cardiac event (CA or syncope), 13 (54%) of S5–Pore–S6 carriers experienced cardiac events, a significantly greater historical event rate as compared to all other regions, except CR-2 (*P* < 0.05) (*Figure 3C*). These findings of an excess of clinical events related to S5–Pore–S6 variants are similar to those reported by Moss *et al*.^13^

## Discussion

Interpreting rare genetic variation in disease-causing genes represents the Achilles heel of clinical genetic testing. This challenge exists because of the now well-recognized observation that rare genetic change is common in humans and most often benign, even for genes known to be associated with disease phenotypes. The relevance of accurate interpretation of rare variants to the appropriate care of patients and families cannot be overstated, as interventions exist that may be life-saving. On the other hand, misinterpreting a more likely benign variant as clinically relevant may lead to unnecessary interventions and anxiety for both patients and their families. The now common approach of whole exome sequencing in diagnostic genetics for a multitude of diseases extends the challenge of rare variant interpretation, as current guidelines recommend the sharing of secondary findings in genes associated with conditions with a risk of significant morbidity or mortality.^14^ The common causative genes for LQTS (*KCNQ1*, *KCNH2*, *SCN5A*) are among the actionable ones for reporting secondary findings.^15^

In this study, to better understand how to interpret secondary findings of variants in *KCNQ1*, we utilized currently published large cohorts of LQTS patients with reported genetic variants in *KCNQ1*, and the large gnomAD public database representing an unselected population with *KCNQ1* genetic variants to determine whether missense variants within specific topological regions of the *KCNQ1* encoded Kv1.7 potassium channel may provide predictive power for the likelihood of disease. Uniquely, we refined this analysis to domain-specific regions in lieu of just considering major regions such as the N-terminus, TLP, and C-terminus as only three distinct zones. Additionally, our current approach allows for a more specific analysis of domains within Kv1.7 that may be most vulnerable to disease-susceptibility when mutated and provides insight to the most relevant structure–function characteristics of the channel.

Our study identifies four specific sub-domains of the *KCNQ1* encoded Kv1.7 channel that are statistically significantly enriched for LQTS type 1 case missense variants at an OR of >2 as compared to the distribution of missense variants from an unselected cohort. These regions include the pore loop of the channel (aa 300–320), the adjacent TM regions comprising the pore (S5, aa 262–282) (S6, 328–348), and a specific region within the C-terminus defined as CR2 in the channel (aa 509–575). The strength of these associations varies, with the pore region (S5–pore loop–S6) having the greatest odds of localizing a missense variant related to the disease. Most interestingly, when variant location within the entire TLP region was compared between cases and controls, significant enrichment for cases was observed, but this was entirely negated when the S5–pore–S6 region was excluded from analysis, indicating that the TLP region outside of the pore region has no greater tendency to missense variants in cases than unselected individuals. The C-terminus. as whole, has a greater likelihood of harbouring variants in an unselected population, with the exception of CR2 within the C-terminus, suggesting this region may have specific functions creating a physiologic vulnerability to disease. Overall, our data is in contrast to previous studies assessing case variant topological enrichment for *KCNQ1* in LQTS type 1.^9,12^ These studies suggested that the enrichment of case variants was inclusive to the entire TLP region. However, neither study assessed sub-domains independently. Our data indicates that the TLP association for enrichment of cases variants is entirely driven by the pore region. In addition, both previous studies implicated large regions of the C-terminus as being more probable for disease variants, but similarly, and in contrast to this study, did not attempt to analyse specific domains that may be driving such an association for a larger region. A noted difference in study analysis was the inclusion of case counts for specific residues in the previous studies, which could skew data analysis implicating large regions for cases due primarily to founder or hot-spot mutations. In clinical practice, hot-spot or founder mutations rarely pose interpretation challenges. In contrast, in our study, each variant was counted once, preventing the risk of false association of sub-domains that may be influenced by large case counts for single mutations. Similar to the results of our data, Millichap and colleagues report a similar clustering of case variant propensity solely in the pore region and CR2 for mutations of *KCNQ2* associated with encephalopathy. Although these investigators also noted enrichment within the S4 TM domain.^16^

### Structure–function relevance

A sub-domain specific approach for case variant enrichment recognizes that various regions within the TM or C-terminus may have specific roles in channel gating, conductance, or tetrameric assembly and that disruption of specific functions may be more or less relevant to provoking clinical disease. In the TM region, the pore region of the channel is critical to ion selectivity and conductance. Functional data supports the vulnerability of this region in substantially altering physiologic behaviour of the channel.^17,18^ Unsurprisingly, this region of Kv1.7 has the strongest enrichment for disease-related missense variants. Although TM regions S1–S4 have a physiological role in voltage sensing and regulating channel gating, particularly S4,^19^ none of these regions were enriched for case variants as compared to variants from an unselected cohort. Similarly, the SAD has long been considered critical to tetrameric channel assembly,^20^ yet an absence of case variant enrichment to this region was also noted. These observations indicate that, as a whole, variants localized to these regions appear as common in cases as in unselected individuals. Somewhat surprising, the C-terminus, when considered in its entirety, was more likely to harbour control variants. However, the specific region most often referred to as CR2 (aa 509–575) showed enrichment for case variants. This particular region of the C-terminal domain contains the distal binding site (aa 508–534) for calmodulin.^21^ Experimental data assessing the interaction of calmodulin with this sub-domain of Kv1.7 has highlighted the importance of calmodulin binding in enabling correct channel folding and assembly, and in channel gating by mediating a calcium-sensitive increase in IKs current.^22,23^ These observations support the importance of CR2 within the C-terminus of Kv1.7 and suggest this region may be as important if not more so for channel assembly as the previously described SAD region.

Our study results, which are based on analysis of large cohorts of cases and an unselected population, and on robust statistical comparisons, offers a novel set of observations that could facilitate the adjudication of missense variants in *KCNQ1*. This is particularly relevant in borderline phenotypes of LQTS, molecular autopsy results where there is an absence of phenotype information, and in rare *KCNQ1* variant observations that may result from secondary findings in WES for other conditions. Although the American College of Molecular Genetics and Genomics has provided a guideline for the interpretation of variants detected in the course of genetic testing, these guidelines are generalized to all genes and do not reflect the nuances of variants that may be gene or disease-specific. In the context of the observations and OR derived from this study, we would propose that rare missense variants localized to the pore loop of *KCNQ1* provide strong evidence (PM1_strong) towards pathogenicity of the variant and that variants within the adjacent S5 and S6 TM regions provoke moderate evidence towards pathogenicity.

Rare variants within CR2 provided a lower OR than the aforementioned sub-domains, but nonetheless was significantly enriched for case missense variants and warrant criteria for supportive evidence towards pathogenicity (PM1_supporting). These additions to variant interpretations for KCNQ1 missense variants could successfully help to upgrade otherwise VUS classifications to LP/P<***>. Other topological regions in this study indicated a more likely enrichment for variants identified in an unselected population, namely the N-terminus and C-terminus (outside of CR-2). While it may be accurate to conclude that variants within these regions as whole may more likely reflect benign variation, criteria to support such a conclusion should be used cautiously given the possibility that specific residues, or hotspot mutations within these regions may have a more profound effect on structure–function.

The availability of large registries of genetic diseases and public databases now provide the opportunity for case–control studies of variant distribution along gene/protein topology. Importantly, as shown is this study, lumping of large gene regions may incorrectly implicate regions as enriched for case variants, while only specific functional domains may be driving such conclusions. Further, consideration of structure–function in relation to variant localization may also provide insight into clinical risk and guide patient management.^24,25^ Guidelines and criteria to assist in the interpretation of rare variants are of importance in guiding clinicians, geneticists, and genetic counsellors. However, the ultimate responsibility and conclusion of variant relevance should be made in the context of robust clinical phenotyping by experienced cardio-genetic clinics with a high volume of experience for uncommon inherited conditions.

### Study limitations

In this study, we used as reference cohort from the gnomAD database v2.1.1 that were void of phenotype information in regard to QT interval. A more ideal control cohort would include subsequent clinical phenotype information. Additionally, sub-domain regions that were not associated with an enrichment of case variants may still harbour specific mutations that are recurring (founder mutations). However, such variants would readily be interpreted towards likely pathogenicity based on ACMG criteria (PS2, PS3, PM2, PM5, PM6, PP1, PP3) recognizing numerous independently reported cases. We did not do a direct comparison of ultra-rare population variants from diagnosed cases vs. the unselected cohort, but of the 311 variants (any MAF) within the unselected cohort, 95% had a MAF < 1 × 10^−4^. Lastly, although our data suggests a more severe clinical presentation with S5–Pore–S6 variants, our cohort size was relatively small and necessitates a larger sample size for validation.

## Conclusion

Our findings demonstrate that specific regions of the TM domain of Kv7.1, comprising the pore and adjacent TM segments (S5 and S6), but not within the remaining two-thirds of the TM region (S1–S4), are enriched for rare missense variants associated with LQTS. An additional region within the C-terminus also demonstrates a higher likelihood of disease-related variants, and contains a known binding site for calmodulin. These data will enhance missense variant interpretation in the *KCNQ1* gene, in the context of secondary findings, and shed light on the most pertinent structural–functional domains in the Kv1.7 potassium channel. Our unique method of analysis may be useful for other genes to help inform interpretation of secondary findings.

## Translational perspective

Secondary findings are a more common observation with the advent of whole exome sequencing. However, the interpretation of genetic variants is still a challenging process. Topological studies identify regions most vulnerable to disease-susceptibility when mutated, assisting in the interpretation of variants identified in unselected individuals.

## Supplementary Material

euad317_Supplementary_Data

## Data Availability

The data underlying this article are available in the article and in its online supplementary material.
