# Supplementary material for: Enhancing the interpretation of genetic observations in KCNQ1 in unselected populations: relevance to secondary findings
Source: Europace. 2023 Oct 28;25(11):euad317. doi: 10.1093/europace/euad317 (PMC10637310; doi:10.1093/europace/euad317)
Supplement: euad317_Supplementary_Data [file euad317_supplementary_data.docx]

**Supplementary Table 1. List of KCNQ1 missense variants**

| **AA Consequence- Cases** |  | **AA Consequence-Controls** |
| --- | --- | --- |
| Y111C |  | A71T |
| P117L |  | A71S |
| C122Y |  | A71V |
| F127L |  | P73T |
| V133I |  | P73S |
| L134P |  | A76T |
| L137F |  | S77F |
| T144A |  | P81L |
| E146K |  | S86R |
| F157C |  | D88E |
| E160K |  | P89T |
| E160V |  | P89L |
| V162M |  | V91L |
| G168R |  | S92P |
| V172M |  | I93V |
| V173D |  | T96R |
| R174C |  | R98H |
| R174H |  | P99R |
| R174P |  | P99Q |
| W176R |  | T104A |
| A178T |  | T104I |
| A178P |  | G108S |
| G179S |  | R109C |
| Y184H |  | R109L |
| Y184S |  | V110I |
| G186R |  | Y111C |
| G189R |  | V129I |
| G189E |  | I132L |
| R190W |  | V133I |
| R190L |  | L137F |
| R190Q |  | L137P |
| R192P |  | S143Y |
| A194P |  | E146K |
| R195P |  | E146Q |
| R195W |  | E146G |
| I198V |  | Q147R |
| S199A |  | T153M |
| D202N |  | V162M |
| D202H |  | V165M |
| I204F |  | F166V |
| **AA Consequence- Cases** |  | **AA Consequence-Controls** |
| I204M |  | G168R |
| S209F |  | G168R |
| V215M |  | T169M |
| T224M |  | V172M |
| S225L |  | V172E |
| R231C |  | R174C |
| R231H |  | R174H |
| Q234R |  | L175I |
| I235N |  | A178T |
| L239P |  | R181C |
| V241G |  | V185M |
| D242N |  | V185L |
| R243C |  | L187F |
| T247N |  | L187P |
| W248R |  | W188G |
| L250P |  | W188S |
| V254L |  | W188C |
| V254M |  | R190W |
| H258N |  | R190Q |
| H258R |  | R190L |
| R259C |  | R192C |
| R259H |  | R192H |
| R259L |  | A194T |
| E261Q |  | R195W |
| E261K |  | R195Q |
| L262V |  | P197L |
| L266P |  | I198V |
| I268S |  | I198T |
| G269S |  | D202N |
| G269D |  | V205M |
| G272D |  | V206L |
| L273F |  | V207M |
| L273R |  | V207L |
| S276F |  | V207L |
| S277P |  | A208V |
| S277W |  | M210I |
| S277L |  | C214Y |
| V280E |  | V215M |
| Y281C |  | V215G |
| L282P |  | G219E |
| A283G |  | Q220K |
| A287E |  | T224M |
|  |  |  |
| **AA Consequence- Cases** |  | **AA Consequence-Controls** |
| G292D |  | S225L |
| R293C |  | R231C |
| A300T |  | R231H |
| A302V |  | L233P |
| A302T |  | Q234H |
| A302E |  | M238V |
| L303P |  | M238L |
| W304R |  | H240R |
| W305S |  | V241I |
| W305R |  | D242N |
| G306R |  | R243C |
| V308D |  | R243H |
| T309R |  | R249S |
| V310I |  | G252R |
| T311I |  | S253P |
| T312I |  | S253A |
| I313M |  | I257V |
| G314C |  | H258Y |
| G314S |  | R259C |
| G314D |  | R259H |
| Y315S |  | Y267C |
| Y315C |  | I268V |
| G316V |  | G269S |
| G316E |  | F270S |
| P320S |  | G272S |
| P320H |  | G272D |
| T322M |  | L273V |
| T322A |  | L273F |
| G325R |  | I274V |
| F339Y |  | A287S |
| A341G |  | A287T |
| A341E |  | A287E |
| A341V |  | E290K |
| L342F |  | G292D |
| P343R |  | R293C |
| P343L |  | R293H |
| A344V |  | V294M |
| S349P |  | G297R |
| G350R |  | G297S |
| F351S |  | G297D |
| L353P |  | S298N |
| K354R |  | A300T |
|  |  |  |
| **AA Consequence- Cases** |  | **AA Consequence-Controls** |
| R360T |  | A302V |
| R360M |  | W305S |
| K362R |  | W305L |
| N365H |  | T311I |
| R366W |  | V319L |
| R366Q |  | T322M |
| A372D |  | T327A |
| L374H |  | T327S |
| W379G |  | A329T |
| R380S |  | A336S |
| R380W |  | F340S |
| E385K |  | F351L |
| S389P |  | K358T |
| K393M |  | K362R |
| K398R |  | F364L |
| D446E |  | R366Q |
| P448L |  | A370V |
| R451W |  | L374H |
| G460S |  | A378T |
| P477L |  | C381Y |
| R511W |  | E385K |
| R518Q |  | N386K |
| R518G |  | P387T |
| R518P |  | D388N |
| M520R |  | D388H |
| Y522S |  | T391A |
| V524G |  | K393N |
| A525T |  | I394L |
| A525V |  | Y395C |
| R533W |  | R397G |
| R539W |  | R397W |
| R539Q |  | R397Q |
| V541I |  | A399S |
| E543K |  | A399V |
| S546L |  | A399G |
| Q547R |  | R401W |
| G548D |  | R401Q |
| V554A |  | P408A |
| R555S |  | P412S |
| R555C |  | K413R |
| R555H |  | V416M |
| K557E |  | V418I |
|  |  |  |
| **AA Consequence- Cases** |  | **AA Consequence-Controls** |
| S566P |  | K420E |
| S566Y |  | K421N |
| S566F |  | K422T |
| I567S |  | K422R |
| I567T |  | K424T |
| G568R |  | L425V |
| K569E |  | N429S |
| S571L |  | V431L |
| L572P |  | T432A |
| F573L |  | T432I |
| R583H |  | P433A |
| N586D |  | G434R |
| T587M |  | P441S |
| G589D |  | H442R |
| A590T |  | T444K |
| R591C |  | T444M |
| R591H |  | D446N |
| R594Q |  | D446E |
| R594P |  | D446E |
| E596K |  | P447H |
| D611N |  | P448S |
| L619M |  | P448Q |
| G626S |  | P448R |
| G635R |  | P448L |
|  |  | E449K |
|  |  | E450K |
|  |  | R451Q |
|  |  | R452W |
|  |  | R452Q |
|  |  | R452L |
|  |  | H455R |
|  |  | H455Q |
|  |  | F456L |
|  |  | D459N |
|  |  | D459V |
|  |  | G460S |
|  |  | G460C |
|  |  | G460D |
|  |  | D462N |
|  |  | S463T |
|  |  | S464P |
|  |  | K467R |
|  |  | **AA Consequence-Controls** |
|  |  | S468G |
|  |  | S468N |
|  |  | L472P |
|  |  | E473Q |
|  |  | M476V |
|  |  | P477T |
|  |  | H478Y |
|  |  | M480T |
|  |  | T482A |
|  |  | T482S |
|  |  | T482N |
|  |  | F485S |
|  |  | A486T |
|  |  | E487K |
|  |  | D488E |
|  |  | G493A |
|  |  | T495A |
|  |  | T495S |
|  |  | L497P |
|  |  | P499S |
|  |  | I500V |
|  |  | I500L |
|  |  | T501A |
|  |  | Q505R |
|  |  | R507W |
|  |  | R507Q |
|  |  | E508G |
|  |  | H509R |
|  |  | H509Q |
|  |  | H510Y |
|  |  | H510R |
|  |  | R511Q |
|  |  | T513A |
|  |  | T513S |
|  |  | R518Q |
|  |  | R519C |
|  |  | R519H |
|  |  | A525T |
|  |  | K526E |
|  |  | K526Q |
|  |  | R533Q |
|  |  | R539W |
|  |  | **AA Consequence-Controls** |
|  |  | V541I |
|  |  | Q544E |
|  |  | G548S |
|  |  | L552F |
|  |  | R555C |
|  |  | R555S |
|  |  | R555H |
|  |  | R555L |
|  |  | D564N |
|  |  | S571P |
|  |  | F573L |
|  |  | I574V |
|  |  | V576I |
|  |  | S580G |
|  |  | S580N |
|  |  | R583C |
|  |  | R583H |
|  |  | G584S |
|  |  | S585N |
|  |  | G589S |
|  |  | G589D |
|  |  | A590T |
|  |  | R591C |
|  |  | N593S |
|  |  | R594Q |
|  |  | V595L |
|  |  | T600M |
|  |  | A607T |
|  |  | D611N |
|  |  | D611Y |
|  |  | G621S |
|  |  | G621C |
|  |  | G622S |
|  |  | P625R |
|  |  | G626S |
|  |  | G628S |
|  |  | G628D |
|  |  | G629S |
|  |  | P630T |
|  |  | P630S |
|  |  | P631R |
|  |  | G635R |
|  |  | **AA Consequence-Controls** |
|  |  | Q640L |
|  |  | P641L |
|  |  | G643S |
|  |  | G646S |
|  |  | V648I |
|  |  | D649N |
|  |  | D649G |
|  |  | F653Y |
|  |  | N657S |
|  |  | T658N |
|  |  | P660S |
|  |  | E663K |
|  |  | V667M |
|  |  | R669T |
|  |  | R669S |
|  |  | R670K |
|  |  | G671S |
|  |  | D673N |
|  |  | E674K |

**Supplementary Table 2. KCNQ1 topology organization**

| **Sub-domains** | **Start** | **End** |
| --- | --- | --- |
| N-terminus | 71 | 121 |
| S1 | 122 | 142 |
| S1/S2 | 143 | 147 |
| S2 | 148 | 168 |
| S2/S3 | 169 | 196 |
| S3 | 197 | 217 |
| S3/S4 | 218 | 225 |
| S4 | 226 | 248 |
| S4/S5 | 249 | 261 |
| S5 | 262 | 282 |
| S5/Pore | 283 | 299 |
| Pore | 300 | 320 |
| Pore/S6 | 321 | 327 |
| S6 | 328 | 348 |
| C-terminus | 349 | 676 |
| TLP | 122 | 348 |
| SAD | 589 | 620 |
| Helix A | 370 | 389 |
| Helix B | 506 | 532 |
| Helix C | 548 | 562 |
| Helix D | 588 | 622 |
| Conserved Region 1 | 349 | 391 |
| Conserved Region 2 | 509 | 575 |
| Conserved Region 3 | 585 | 607 |

TLP= transmembrane/linker/pore spanning region
